# Putting pain into words: the psalms of lament as an aid for the alexithymic

**DOI:** 10.3389/fpain.2025.1659170

**Published:** 2025-12-08

**Authors:** Emmanuel Bäckryd

**Affiliations:** Pain and Rehabilitation Center, and Department of Health, Medicine and Caring Sciences, Linköping University, Linköping, Sweden

**Keywords:** alexithymia, existential, lament, pain, suffering

## Abstract

This paper explores how existentially and religiously laden texts such as the psalms of lament can help alexithymic persons living with chronic pain to articulate their emotions. It is proposed that verbalizing emotions through psalms of lament can promote coping, and this hypothesis is exemplified by a reading of Psalm 13. The first part of Psalm 13 helps the alexithymic reader turn existential anguish into questions, expressing feelings of being forgotten, ignored, worried, and antagonized. In the second part, there is a shift towards expressing expectations using the imperative mood, highlighting assertiveness. Assertiveness, though not part of alexithymia, relates to emotional expressiveness, and learning to express requests and expectations is health-promoting. In the third part, hope is introduced. This is linked to the psalmist's ability to say “I.” Self-assertion is crucial for overcoming externally-oriented thinking, empowering persons to see themselves as active agents. Trust and hope are active stances, essential for living well despite chronic pain. Hence, texts viewed as sacred by alexithymic readers can help them get a language with which to express their discomfort.

## Introduction

It has been claimed that about a tenth of the population is alexithymic (1). Simply put, the construct of alexithymia designates problems with putting emotions into words. The Toronto Alexithymia Scale (TAS-20), a questionnaire that is often used to assess alexithymia (2, 3), consists of three subscales, illustrating three important facets of the construct: *difficulty identifying feelings*; *difficulty describing feelings*; and *externally-oriented thinking*, i.e., the tendency of individuals to focus their attention externally and not on the inner emotional experience (4). Using the international threshold criterion for alexithymia (TAS-20 sum score ≥61), it has been estimated that the prevalence in the general population is 10 percent (5). There seems to be an association between this construct and male gender, increasing age, low educational level, poor perceived health, and depression (1).

Pain is defined by the International Association for the Study of Pain (IASP) as “an unpleasant sensory and emotional experience associated with, or resembling that associated with, actual or potential tissue damage” (6). Pain is hence by definition both a sensory and an emotional experience. In the present paper, the emotional component of pain is in the foreground. Needless to say, this focus does not in any way invalidate the very real effect that pain has on the lived experience of patients. Neither does it reduce pain to an irrational feeling without reference to the real world. Chronic pain is real, and because it is real, it has an emotional component. In this context, it is interesting to reflect on the relationship between pain as an emotion and difficulties in putting this emotion into words. Alexithymia, literally meaning no words for emotions (*a* = lack, *lexis* = word, *thymos* = emotions), has been described as a prominent feature in chronic pain conditions (7, 8). Hence, the alexithymia construct is not only theoretically or interpersonally relevant; it also seems important from a health perspective in general, and perhaps for chronic pain in particular.

About 20% of the adult population suffers from chronic pain (9). Chronic pain can often be viewed as a disease in its own right rather than just a symptom of another underlying disease or injury. Given the present status of medical science, such chronic pain is very difficult to treat pharmacologically (10). Research is ongoing concerning the pathophysiology of different chronic pain conditions (11), the long-term vision being that it might become possible in the future to prescribe effective analgesics for chronic pain using valid and reliable biomarkers (12). Meanwhile, patient education, psychotherapy, and judicious physical activity are central to evidence-based chronic pain rehabilitation. Simply put, if it is not possible to take away the pain, it is still possible to help patients live meaningful lives despite pain. In such circumstances, not being able to identify and put your emotions into words is probably a drawback. In a broader context, Pennebaker's work on expressive writing (13) has put the benefits of emotional expression on the agenda of medical research, e.g., concerning the psychological well-being of cancer patients (14).

Believers have for centuries used the Book of Psalms in prayer and devotion. Between one third to one half of the 150 psalms are laments (15), and Psalm 13 has been characterized as being a typical psalm of lament (16). Generally speaking, in lament, “Everything in on the table: doubt, anger, despair, guilt, resentment. There is no requirement of politeness. There is no need of gentility” (15). Typical for lament is also “a distinctive movement from plea to praise” which is “an act of boldness” in the sense that lament thereby entails the possibility of change and transformation (15). In that sense, lament is dynamic, it is a movement, an active involvement not only with God but with the world and the circumstances that the believer faces. It has been suggested that biblical psalms of lament can have an important function as means of coping with trauma (17), or resolving anger towards God (18). Even the imprecatory psalms, which are psalms that describe feelings of hate and animosity, have been said to be useful in the sense that they “provide a valuable mechanism for the cathartic release of negative emotion” (19). All in all, it has been proposed that the psalms, many of which were shaped thousands of years ago by traumatic circumstances such as war and deportation, can serve as religious coping means that believers may draw upon in the present when facing trauma and adversity (17).

The aim of this paper at the interface of reader-response studies and medicine is to show how a psalm of lament such as Psalm 13 might help alexithymic believers, who suffer from for instance chronic pain, to put their existential emotions into words. In doing so, I will assume that such verbalization is helpful and promotes coping. I will also assume a formative use of biblical texts, i.e., I will assume that a putative believer uses such texts in devotions and spiritual practices aiming at character formation (c.f., the tradition of *lectio divina*). Importantly, this paper is hypothesis-generating and theoretical rather than empirical, i.e., the hypothesis that is here described needs to be tested in empirical and confirmatory studies with an appropriate methodology, the elaboration of which is outside the scope of the present paper.

## Methods

This is not an exegetical paper. The original text, its Hebraic wording and meaning is not the focus of this study. Rather, this work is akin to a reader-response approach to biblical texts (20), focusing on the way putative alexithymic readers might be helped by reading Psalm 13. Hence, I will simply use the translation of New International Version (NIV):

^1^How long, LORD? Will you forget me forever?

How long will you hide your face from me?

^2^How long must I wrestle with my thoughts

and day after day have sorrow in my heart?

How long will my enemy triumph over me?

^3^Look on me and answer, LORD my God.

Give light to my eyes, or I will sleep in death,

^4^and my enemy will say, “I have overcome him,”

and my foes will rejoice when I fall.

^5^But I trust in your unfailing love;

my heart rejoices in your salvation.

^6^I will sing the LORD’s praise,

for he has been good to me.

A superficial analysis of this short psalm reveals a threefold division consisting of two verses each. It will be argued that each part can be hypothesized to be beneficial for alexythemic believers who try to put their existential anguish into words, for instance in a chronic pain setting.

### Turning existential anguish into questions

Verses 1-2 express four strong human emotions: being forgotten; being ignored; being worried; being antagonized. Of course, the well-known parallelistic tendency of biblical poetry must be taken into account. Hence, the twin ideas in verse 1 of being forgotten and being ignored are in many ways synonymous (i.e., synonymous parallelism). Being forgotten conveys a feeling of geographical distance, God being portraited as a relative or friend on a journey far away who never sends any news. On the other hand, the feeling of being ignored, i.e., that God's face is hidden, assumes proximity in space—it is as if the psalmist views him- or herself in the same room as God but without being able to see the Face. The God who is present is nonetheless invisible, and the psalmist feels ignored. “I know you are there, so why do you hide?” Feeling ignored is a powerful emotion, and there is neuroscientific evidence suggesting that social exclusion activates a network of brain neurons that are also involved in the experience of pain (21). If God is in any sense “real” to the believer, it seems reasonable to hypothesize that the ability to somehow overcome this sense of isolation and “reconnect” to God might be helpful. Being able to put one's existential questions into words can be seen as a first step, and psalms of lament can be used to that effect by believers—and perhaps particularly by alexithymic believers. In many ways, verse 1 is about the actualization in real life of what philosophers of religion call “the hiddenness of God” (22). This “problem” is reminiscent of, but partly different from, the problem of evil in the philosophy of religion.

In verse 2, worry is expressed—worry in general, but also in particular worry about “enemies”. Applied to a pain medicine context, it is well-known that psychological distress is common in chronic pain patients, and a subset of them also express what could be characterized as social distress—e.g., reporting low levels of social support and high levels of punishing responses from significant others (23). Metaphorically and congruent with a reader-response use of the Bible, this verse could hence help alexithymic believers to put both pain-related psychological and pain-related social distress into words.

All in all, verses 1-2 can help believers turn their anguish into tough existential questions addressed to the God they believe in. Indeed, biblical spirituality, as expressed paradigmatically by the attitude of the suffering Job, does not shy away from asking tough existential questions in prayers and devotions—as illustrated by these four questions that all begin by “How long?” The implication is that suffering has been taken place for too long, a situation that chronic pain patients by definition can identify themselves with (24). The alexithymic believer can here find words in which his or her emotion can be clothed. There seems to be some empirical support for the notion that chronic pain patients more often than others feel abandoned by God (25).

### Expressing existential expectations by using the imperative mood

In NIV, verses 1-2 are questions, whereas verses 3-4 express expectations by using the imperative mood. Spirituality is not only about asking questions, it can also be about using the imperative mood in prayer and devotion. Hence, the psalmist is here very assertive. Interestingly, alexithymia and assertiveness have been described as “two indirect measures of the difficulties in emotional expressiveness” (26). Hence, although assertiveness is not strictly speaking part of the concept of alexithymia, it is nonetheless a related concept. As expressed by Duckworth and Mercer (27):

Assertive behavior usually centers on making requests of others and refusing requests made by others that have been judged to be unreasonable. Assertive behavior also captures the communication of strong opinions and feelings. Assertive communication of personal opinions, needs and boundaries has been defined as communication that diminishes none of the individuals involved in the interaction, with emphasis placed on communication accuracy and respect for all persons engaged in the exchange.

A low capacity for assertiveness is problematic (28, 29). For instance, there is some evidence to support the notion that assertiveness might protect against burnout (30). All in all, learning to express requests and expectations might be viewed as health-promoting, and verses 3-4 can help believers do just that when they turn the words of the psalmist into their own. Hence, Psalm 13 can help the suffering believer to state his or her wishes frankly—directly into God's “face”, but also, as a consequence, to humans who act like “enemies” (verse 4).

### Hope and being able to say “I”

Now to verses 5-6. Here, in the final third part of the text, hope emerges, and this hope is associated with the psalmist's capacity to say “I”. Commenting on verse 6, Old Testament exegete Helmer Ringgren says that the “I” is here emphasized, i.e., it could therefore be translated “I, for one” (16). Of course, there seems to be a link between the capacity to say “I” and being able to be assertive (c.f. verses 3-4). Another side of assertiveness is being able to say “I”, i.e., asserting yourself and your point of view in a balanced way can be viewed as “the behavioral middle ground, lying between ineffective passive and aggressive responses” (27). Moreover, learning to say “I” can also be part of overcoming one of the three facets of the concept of alexithymia, namely externally-oriented thinking. Learning to say “I” shifts the focus from only a description of circumstances to a vision of the self as an agent capable of having a say-so on life. Hence, there is something empowering in the ability to say “I” and, in the case of the psalmist, this is parallelled by the rise of hope. Trusting in God is not to be equated with passivity. Trust and hope are active stances grounded in healthy self-awareness. Applied to the chronic pain setting, one is reminded of the importance of a rehabilitative stance, whereby the patient is not a mere passive recipient of biomedical interventions but instead is an active agent whose “work” (not least psychologically) is essential for the ability to live a good life despite the pain that present medical science is not able to alleviate.

## Conclusion

I have argued that Psalm 13, if used in devotions and spiritual practices in the *lectio divina* tradition, can help the alexithymic believer who suffers from chronic pain (a) to put deeply human emotions into words by asking questions (verse 1-2), (b) to assert oneself by using the imperative mood (verses 3-4), and (c) to put hope and trust into words by use of the personal pronoun “I” (verses 5-6). Of course, Psalm 13 is merely one example of the rich tradition of psalms of lament. Also, the example of chronic pain has been chosen due to the profession of the author; if the basic contention of the paper is right, there is nothing that hinders a wider application of the principles described in it. All in all, viewed from the perspective of a formative reading of the Bible, the psalms of lament can be viewed as effective forms of emotional expression that can help the alexithymic believer get a language with which to express his or her discomfort.

## Data Availability

The original contributions presented in the study are included in the article/Supplementary Material, further inquiries can be directed to the corresponding author.
